# Curcumin Exerts Antinociceptive Effects in Cancer-Induced Bone Pain via an Endogenous Opioid Mechanism

**DOI:** 10.3389/fnins.2021.696861

**Published:** 2021-09-03

**Authors:** Guanghai Zhao, Yongqiang Shi, Chaoyang Gong, Taicong Liu, Wei Nan, Lin Ma, Zuolong Wu, Chaoming Da, Kaisheng Zhou, Haihong Zhang

**Affiliations:** ^1^Department of Orthopedics, Lanzhou University Second Hospital, Lanzhou, China; ^2^The Second Clinical Medical College, Lanzhou University, Lanzhou, China; ^3^Orthopaedics Key Laboratory of Gansu Province, Lanzhou University, Lanzhou, China

**Keywords:** curcumin, antinociception, cancer-induced bone pain, endogenous opioid peptides, mechanism

## Abstract

Cancer pain is one of the main complications in advanced cancer patients, and its management is still challenging. Therefore, there is an urgent need to develop novel pharmacotherapy for cancer pain. Several natural products have attracted the interest of researchers. In previous studies, curcumin has proved to exhibit antitumor, antiviral, antioxidant, anti-inflammatory, and analgesic effects. However, the analgesic mechanism of curcumin has not been elucidated. Thus, in this study, we aimed to elucidate the antinociceptive potency and analgesic mechanism of curcumin in cancer-induced bone pain. Our results showed that consecutive curcumin treatment (30, 60, 120 mg/kg, i.p., twice daily for 11 days) produced significant analgesic activity, but had no effect on the progress of the bone cancer pain. Notably, pretreatment with naloxone, a non-selective opioid receptor antagonist, markedly reversed the antinociceptive effect induced by curcumin. Moreover, in primary cultured rat dorsal root ganglion (DRG) neurons, curcumin significantly up-regulated the expression of proopiomelanocortin (*Pomc*) and promoted the release of β-endorphin and enkephalin. Furthermore, pretreatment with the antiserum of β-endorphin or enkephalin markedly attenuated curcumin-induced analgesia in cancer-induced bone pain. Our present study, for the first time, showed that curcumin attenuates cancer-induced bone pain. The results also suggested that stimulation of expression of DRG neurons β-endorphin and enkephalin mediates the antinociceptive effect of curcumin in pain hypersensitivity conditions.

## Introduction

Cancer pain, considered to be a major complication of many advanced malignancies, including lung, breast, and prostate cancers, severely affects patients’ quality of life (Mercadante, 1997; Portenoy and Lesage, 1999; Delaney et al., 2008; Breivik et al., 2009). According to the statistics issued by a recent study, there are approximately two-thirds of patients with advanced and metastatic cancer suffering from pain (van den Beuken-van Everdingen et al., 2016). At present, opioid analgesics are still the most common used drugs in the treatment of cancer pain. However, several opioid-related side effects, such as tolerance, constipation, and respiratory depression, adversely limited its clinical utility (Bruera and Kim, 2003). In addition, morphine has been reported to promote tumor metastasis through immunosuppression and angiogenesis (Afsharimani et al., 2011). Therefore, the development of new pharmacotherapy for alleviating cancer pain is one of the greatest social and clinical needs.

Natural products have shown promising prospects in the treatment of pain. Morphine, one of the first natural compounds extracted from *Papaver somniferum*, is still known as the classic analgesic. Ziconotide is the synthetic equivalent of a naturally occurring conopeptide found in marine snail (*Conus magus*), and it has been approved on December 28, 2004 in the United States as a treatment for severe chronic pain (Wallace, 2006). Therefore, natural products are considered excellent candidates in the research and development of new analgesic drugs.

Curcumin is a phenolic compound isolated from *Turmeric* with small molecular weight, and has been proved to exert antitumor, antiviral, antioxidant, anti-inflammatory, and other pharmacological activities (Maheshwari et al., 2006). Clinical evidence suggests that curcumin has beneficial effects against various human diseases, such as osteoarthritis, solid tumors, uveitis, and benign prostatic hyperplasia (Mirzaei et al., 2017). Notably, several studies revealed that curcumin exerts significant analgesic effect in various preclinical pain models. For example, both intrathecal and intraperitoneal injections of curcumin can alleviate complete Freund’s adjuvant (CFA)-induced inflammatory pain (Chen et al., 2015). Repeated systemic treatment of curcumin reduced neuropathic pain induced by peripheral nerve injury, diabetes, and chemotherapy drugs (Greeshma et al., 2015; Liu et al., 2016a; Jia et al., 2017; Zhang et al., 2018a). Moreover, oral administration of curcumin exhibited significant antinociceptive effect and promoted recovery in a rat model of postoperative pain (Zhu et al., 2014). Furthermore, studies have shown that the analgesic effects of curcumin are associated with the opioid system (Zhao et al., 2012; Banafshe et al., 2014). However, there has been no direct evidence on the interaction between curcumin and the endogenous opioid system.

Therefore, in this study, we aimed to access the analgesic effect of curcumin in a mouse model of cancer-induced bone pain. We also explored the underlying mechanisms of the antinociceptive effect of curcumin. To our knowledge, our present study is the first to reveal that curcumin mediates the expression of endogenous opioid peptides to produce analgesic effects in cancer-induced bone pain.

## Materials and Methods

### Experimental Animals

Male C57BL/6 mice (20–25 g) were purchased from the Experimental Animal Center of Lanzhou Veterinary Research Institute, and newborn Wistar rats (12 days old) were obtained from the Experimental Animal Center of Lanzhou University. The adult mice were housed in the animal room with a 12-h light-dark cycle and climate-controlled at a 22–24°C environment, with freely available food and water. The experimental procedures were approved by the Ethics Committee of Lanzhou University, and were conducted following the European Community guidelines for the use of experimental animals (2010/63/EU). All behavioral tests are conducted from 8:00 a.m. to 6:00 p.m., and the testers were unaware of the treatment of the mice.

### Drugs and Reagents

Curcumin was purchased from Solarbio (Beijing, China) with 95% purity. Naloxone, β-Funaltrexamine (β-FNA), nor-binaltorphimine (nor-BNI), and naltrindole (NTI) were purchased from Sigma-Aldrich (St. Louis, MO, United States). β-endorphin antiserum and enkephalin antiserum were purchased from Abcam (Cambridge, United Kingdom). Curcumin was dissolved in a vehicle with 5% dimethyl sulfoxide (DMSO), 10% Cremophor EL, and 85% saline. Other drugs were dissolved in saline.

### Mice Model of Bone Cancer Pain

A mouse model of bone cancer pain was established as previously reported (Gonzalez-Rodriguez et al., 2017). Briefly, mice were anesthetized with intraperitoneal injection of pentobarbital sodium (20 mg/kg). Next, the right tibial plateau was exposed, and B16-F10 melanoma cells (1 × 10^5^, 5 μl) were injected into the tibia cavity using a microsyringe. The injection site was then sealed with sterile medical glue to prevent the cancer cells from flowing out. Finally, the wound was closed using surgical suture, and erythromycin ointment was applied to the sutured wound to prevent infection. After recovering from anesthesia, the mice were housed separately.

### Drug Administration

The dosage of curcumin used for experiment was 30, 60, and 120 mg/kg. From the first day after surgery, curcumin at different concentrations or vehicle was administered (i.p.) twice daily until the 11th day. A non-selective antagonist (naloxone) or selective antagonist (NTI, β-FNA, or nor-BNI) of the opioid receptor was administered (i.p.) at 10 min, 10 min, 4 h, and 30 min before the injection of vehicle or curcumin, respectively. β-endorphin antiserum and enkephalin antiserum were administered (i.pl.) at 30 min before curcumin injection. The administration time and dosage of the antagonist and antiserum were consistent with those used in previous studies (Tejada et al., 2017; Zhang et al., 2018b).

### Behavioral Assessment of Mechanical Allodynia

In this study, an electrical von Frey filament (38450; Ugo, Italy) was used to test the mechanical allodynia. The mice were placed in a plastic box with a metal grid on the bottom and allowed to acclimatize in this environment for at least 30 min before the behavior test began. The withdrawal threshold was considered to be the lowest force that caused a mouse to shrink its paw when using the von Frey filament to stimulate the hindfoot. The baseline withdrawal threshold of mice was measured before surgery. On Day 3, 5, 7, 9, and 11 after surgery, the paw withdrawal thresholds before administration were determined, and the 4 h post-injection paw withdrawal thresholds were simultaneously measured on Day 5, 7, 9, and 11. We conducted three measurements at each time point, at an interval of approximately 2 min. Opioid receptor antagonistic and selective antagonistic experiments were performed on the 11th day after surgery.

### Primary Cell Cultures

Perform primary culture of DRG neurons as previously reported (Lin and Chen, 2018). Use 12-day-old rats for DRG collection. Sacrifice the rats by decapitation and quickly remove the muscle covering the spine. Then use bone cutters to remove the dorsal part of the vertebrae and expose the spinal cord. DRG can be seen on both sides of the spinal cord. Collect the bilateral DRG with micro-scissors into mixed digestive enzymes with 342 U/ml collagenase I and 3.9 U/ml neutral protease for 45 min. After centrifugation, the cells were evenly planted on poly-D-lysine-coated glass slides containing 10% (v/v) fetal bovine serum, penicillin (100 U/ml), streptomycin (100 μg/ml), and 0.05% nerve growth factor (NGF) in DH10 medium. Next, the cells were placed overnight in an incubator at 37°C with 5% CO_2_ and were starved for 2 h before drug treatment.

### Real-Time Quantitative PCR (RT-PCR)

DRG neurons were incubated with the drug for 6 h and split using Trizol reagent (Accurate Biotechnology, Hunan, China). Total RNA was extracted according to the manufacturer’s protocol (Accurate Biotechnology, Hunan, China), and the RNA concentration was determined using a spectrophotometer. Next, 0.5 μg of the total RNA was reverse-transcribed using a reverse transcription kit (Accurate Biotechnology, Hunan, China). An Agilent MX3005P (Agilent, United States) detection system and SYBR Premix Ex Taq^TM^II kit (Accurate Biotechnology, Hunan, China) were used to perform the RT-PCR. The entire process included three stages: pre-denaturation (95°C, 60 s), amplification (95°C for 5 s, then 60°C for 60 s, 40 cycles), and melting. The primers were 5′- TGC GAC TTC AAC AGC AAC TC -3′ and 5′- CTT GCT CAG TGT CCT TGC TG -3′ for *Gapdh* (Minett et al., 2015); 5′-CTT TCC GCG ACA GAG CCT-3′ and 5′-CCA GCT CCA CAC GTC TAT GG-3′ for the β-endorphin precursor *Pomc* (Mao et al., 2019); 5′- TTC AGC AGA TCG GAG GAG TTG -3′ and 5′- GAA GCG AAC GGA GGA GAG AT -3′ for the enkephalin precursor proenkephalin (*Penk*) (Minett et al., 2015); and 5′-CCT GTC CTT GTG TTC CCT GT-3′ and 5′-AGA GGC AGT CAG GGT GAG AA-3′ for the dynorphin precursor prodynorphin (*Pdyn*) (Mao et al., 2019). Gene expression was standardized according to the expression level of *Gapdh* and analyzed using the 2^–Δ^ ^Δ^ ^CT^ method.

### ELISA

DRG neurons were incubated with drugs for 6 h, and the cell culture fluid was centrifuged (3,000 rpm, 15 min). The concentration of β-endorphin and enkephalin protein was determined using an ELISA kit, according to the manufacturer’s instructions (Blue Gene Life Science Co., Ltd., Shanghai, China). A microplate reader was used to read the absorbance at 450 nm. Data was analyzed using ELISA Calc software and a four-parameter logistic model.

### Statistical Analysis

All data in this study were displayed as means ± S.E.M. and analyzed using GraphPad Prism 8.0.1 (San Diego, CA, United States). Maximum possible effect (MPE) % = (post-drug threshold - pre-drug threshold)/(baseline threshold - pre-drug threshold) × 100%. For behavioral tests, the PWTs between groups were analyzed using one-way ANOVA, followed by Dunnett’s or Bonferroni’s *post hoc* test. For RT-PCR and ELISA results, *t*-test was used to compare differences between groups. The difference was considered significant when *P* < 0.05.

## Results

### Intraperitoneal Administration of Curcumin Exerted an Anti-allodynic Effect Against Cancer-Induced Bone Pain

**FIGURE 1:**
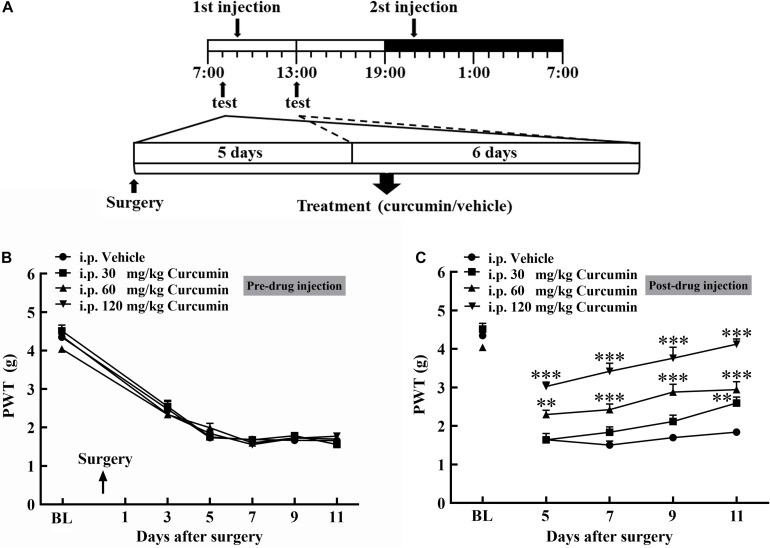
Chronic treatment with curcumin inhibited B16-F10 melanoma cell-induced bone cancer pain behavior in mice in a dose-dependent manner. Schematic of experiment **(A)**. Mice were injected with B16-F10 melanoma cells (1 × 10^5^, 5 μl) and then treated with curcumin (30, 60, and 120 mg/kg, i.p.) twice daily for 11 days starting at 24 h after tumor injection. The withdrawal threshold before the injection was measured on the 3rd, 5th, 7th, 9th, and 11th day **(B)** and after the injection was measured on the 5th, 7th, 9th, and 11th day **(C)**. Data are shown as means ± SEM, *n* = 7 per group. * *P* < 0.05, **P < 0.01, and ***P < 0.001 compared with the vehicle group, one-way ANOVA followed by Dunnett’s test.

A total of 28 male mice with bone cancer pain were divided into four groups and received intraperitoneal administration of the vehicle or curcumin (30, 60, or 120 mg/kg) twice daily. At 5, 7, 9, and 11 days after surgery, the withdrawal threshold was tested pre- and 4 h post-injection of curcumin (Figure 1A). The withdrawal threshold reached a stable state on Day 5 after surgery, and intraperitoneal injection of the vehicle or curcumin (30, 60, or 120 mg/kg) had no significant effect on the withdrawal threshold pre-drug injection (Figure 1B). However, it relieved mechanical allodynia at 4 h after injection on Day 5, 7, 9, and 11 in a dose-dependent manner (Figure 1C). The results showed that curcumin at a dose of 60 and 120 mg/kg exerted significant analgesic effect at 4 h after administration on Day 5, 7, 9, and 11 after surgery. The lowest dose of curcumin (30 mg/kg) produced analgesic effect only on Day 11 after surgery.

### Effects of Opioid Receptor Antagonist and Selective Antagonists on the Analgesic Effect of Curcumin

**FIGURE 2:**
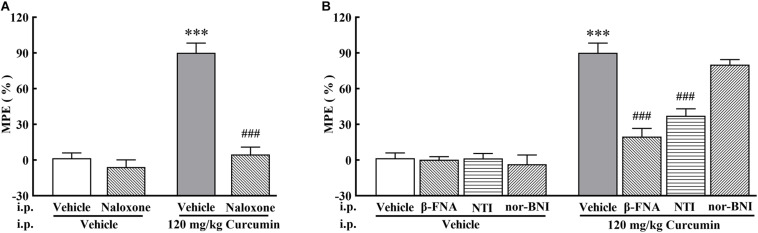
Effect of pretreatment with opioid receptor antagonists on the anti-allodynia activity of curcumin in cancer-induced bone pain mice. Mice were treated with curcumin (120 mg/kg, i.p.) twice a day for 11 days after the injection of B16-F10 melanoma cells (1 × 10^5^, 5 μl). In the 11th day, The opioid antagonists naloxone (1 mg/kg, i.p.) **(A)**, NTI (0.5 mg/kg, i.p.), β-FNA (0.5 mg/kg, i.p.), and nor-BNI (0.5 mg/kg, i.p.). **(B)** were administered at 10 min, 10 min, 4 h, and 30 min prior to curcumin (120 mg/kg, i.p.) injection, respectively. MPE% induced by curcumin (120 mg/kg, i.p.) 4 h after administration was evaluated. Data are shown as means ± SEM, *n* = 6 per group. *P < 0.05, **P < 0.01, and ***P < 0.001 compared with the vehicle + vehicle group, ^###^*P* < 0.001 compared with the vehicle + curcumin group, one-way ANOVA followed by Bonferroni’s test.

To investigate the involvement of the opioid system in curcumin-induced analgesia, the non-selective opioid receptor antagonist naloxone, δ-opioid receptor (DOR) selective antagonist NTI, μ-opioid receptor (MOR) selective antagonist β-FNA, and κ-opioid receptor (KOR) selective antagonist nor-BNI were administered on Day 11 after surgery to bone cancer-bearing mice treated twice daily with curcumin (120 mg/kg, i.p.). The results showed that the curcumin-induced analgesic effect was completely blocked by naloxone (1 mg/kg, i.p.) (Figure 2A). In addition, the MOR selective antagonist β-FNA (0.5 mg/kg, i.p.) and the DOR selective antagonist NTI (0.5 mg/kg, i.p.), but not the KOR selective antagonist nor-BNI (0.5 mg/kg, i.p.), significantly reduced the analgesic effect of curcumin (Figure 2B). The above results indicated that the analgesic effect of curcumin was mainly related to MOR and DOR, but independent of KOR.

### Curcumin Regulated DRG Neurons Expression of Endogenous Opioid Peptide

**FIGURE 3:**
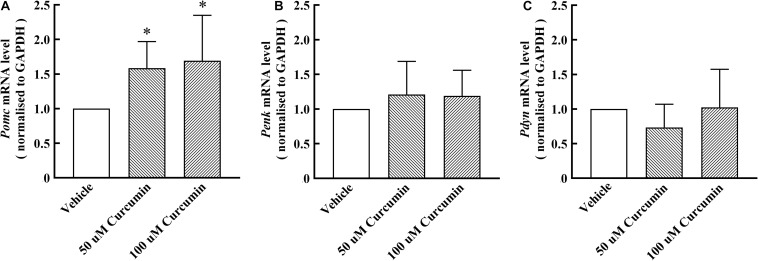
Effect of curcumin on the mRNA levels of endogenous opioid peptides in primary DRG neurons. The cells were collected 6 h after curcumin or vehicle incubation with primary cells, and the expression of *Pomc*, *Penk*, and *Pdyn* was evaluated by using RT-PCR. Both 50 and 100 μM curcumin significantly increased the level of *Pomc* mRNA **(A)** in rat DRG neurons, but had no significant effect on *Penk* and *Pdyn* mRNA **(B,C)**. Data are shown as means ± SEM, *n* = 5–6. *P < 0.05 compared with the vehicle group, *t*-test.

**FIGURE 4:**
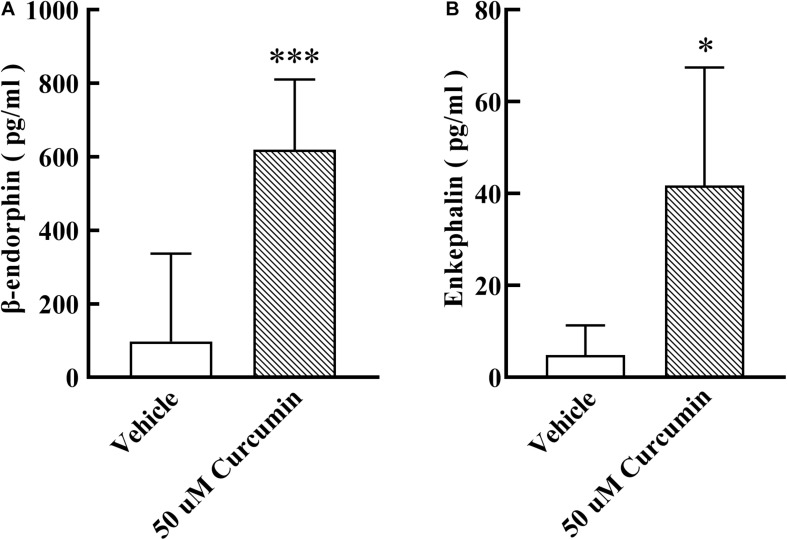
Effect of curcumin on the release of β-endorphin and enkephalin in primary DRG neurons. The culture medium was collected 6h after curcumin incubation with primary cells, and the release of β-endorphin and enkephalin was determined by using an ELISA kit. Curcumin at 50 μM significantly increased the release of β-endorphin **(A)** and enkephalin **(B)**. Data are shown as means ± SEM, *n* = 4–11. *P < 0.05 and ***P < 0.001 compared with the vehicle group, *t*-test.

Based on the results of *in vivo* experiments, we hypothesized that curcumin may exert analgesic effect by promoting the release of endogenous opioid peptides. β-endorphin, enkephalin, and dynorphin, which are considered as classic endogenous opioid peptide members, act as neurotransmitters and neuromodulators at the opioid receptors and produce analgesia (Holden et al., 2005). Their precursors are *Pomc*, *Penk*, and *Pdyn*, respectively. To test its stimulatory effect on the expression of endogenous opioid peptide precursors in cultured primary cells, curcumin (50, 100 μM) was incubated for 6 h with DRG neurons. The gene expression of *Pomc*, *Penk*, and *Pdyn* from each set of cells was measured using RT-PCR. As shown in Figure 3A, treatment with curcumin for 6 h increased the expression of *Pomc* in DRG neurons in a dose-dependent manner. In contrast, curcumin did not significantly change the expression of the *Penk* and *Pdyn* genes in DRG neurons at 50 or 100 μM (Figures 3B,C). In addition, we verified the release of endogenous opioid peptides at the protein level. The effects of curcumin on the release of β-endorphin and enkephalin in DRG neurons were evaluated by ELISA in primary DRG neurons. As shown in Figures 4A,B, exposure of curcumin (50 μM) significantly increased the protein expression of β-endorphin by 6.3-fold and enkephalin by 8.6-fold compared with baseline levels, respectively.

### Effect of β-Endorphin and Enkephalin Antiserum on the Analgesic Effect of Curcumin

**FIGURE 5:**
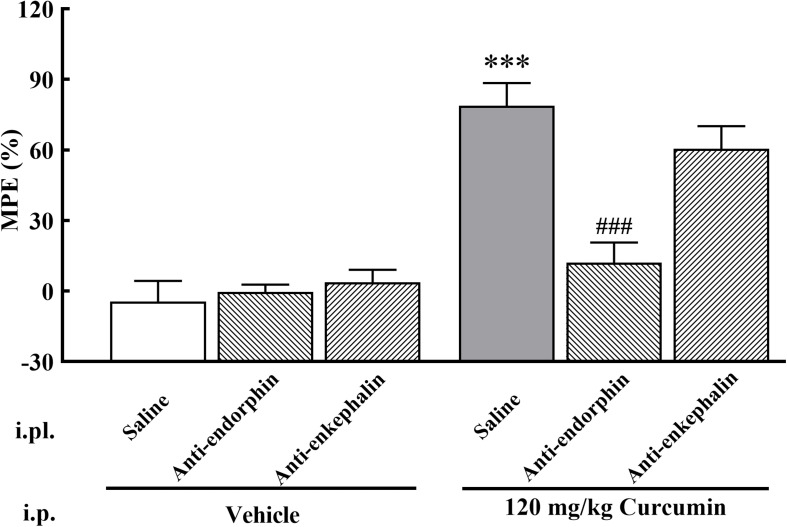
Effect of β-endorphin and enkephalin antiserum pretreatment on the anti-allodynia activity of curcumin in cancer-induced bone pain mice. Mice were treated with curcumin (120 mg/kg, i.p.) twice a day for 11 days after the injection of B16-F10 melanoma cells (1 × 10^5^, 5 μl). On the 11th day, β-endorphin and enkephalin antiserum (1:40 dilution, 20 μl, i.pl.) were administered at 30 min prior to curcumin (120 mg/kg, i.p.) injection, respectively. MPE% induced by curcumin (120 mg/kg, i.p.) 4 h after administration was evaluated. Data are shown as means ± SEM, *n* = 6–7 per group. ***P < 0.001 compared with the saline + vehicle group, ^###^*P* < 0.001 compared with the vehicle + curcumin group, one-way ANOVA followed by Bonferroni’s test.

To assess whether curcumin exhibits analgesic effect by promoting the release of β-endorphin and enkephalin, the antibodies against β-endorphin and enkephalin were employed. A total of 19 male mice with bone cancer pain into three groups and received intraperitoneal administration of curcumin (120 mg/kg) twice daily. The three groups received i.pl. of saline (20 μl), β-endorphin antiserum (1:40 dilution, 20 μl) or enkephalin antiserum (1:40 dilution, 20 μl) followed by curcumin (120 mg/kg) 30 min later. As shown in Figure 5, the administration of β-endorphin antiserum, but not enkephalin antiserum, abolished the analgesic effect in response to mechanical stimulation induced by systemic administration of curcumin.

## Discussion

Because of its close association with movement and weight bearing, bone cancer pain is considered one of the most challenging pain conditions to control (Kane et al., 2015). Strong opioids are the primary treatment for bone cancer pain (Hauser et al., 2017). However, opioid-related side effects, such as addiction, tolerance, constipation, oversedation, and respiration inhibition, adversely limited their clinical application (Guo et al., 2017). At present, several studies have been devoted to developing new analgesics for the treatment of bone cancer pain, but there is still no effective strategy. Thus, in this study, we evaluated the analgesic effect of curcumin intraperitoneal injection twice daily in a bone cancer pain mouse model, which was established via injection of B16-F10 melanoma cells into the tibias of male C57BL/6 mice. Our current results suggested that intraperitoneal injection of curcumin is effective for the management of bone cancer pain. Furthermore, curcumin was shown to exert analgesic effect by promoting the release of endogenous opioid peptides in DRG neurons.

Several studies suggested that curcumin exerts potent analgesic activities in various pain models (Sun et al., 2018). However, the analgesic effect of curcumin in cancer-induced bone pain remains to be investigated. In our present study, we observed that repeated curcumin treatment exerted an acute dose-dependent antinociception on Day 5, 7, 9, and 11 post-surgery, but had no effect on the development of cancer-induced bone pain. In addition, previous studies have shown that repeated curcumin treatment significantly relieved the development of inflammatory pain and neuropathic pain, which is inconsistent with the results obtained in our study (Chen et al., 2015; Liu et al., 2016b). This phenomenon might be associated with the difference between the mechanism of different pain models. Bone cancer pain is a complex pain state that is generally considered to be a superposition of neuropathic and inflammatory pain (Falk and Dickenson, 2014). Tumor cells stimulate the expression of local inflammatory mediators, creating a highly acidic environment, which increases the sensitivity of peripheral nerve endings in the bone marrow and bone matrix. When combined with damage to nerve endings caused by tumor infiltration, the resulting pain is a mixture of inflammation and neuropathic processes. In inflammatory and neuropathic pain, the effect of curcumin may be due to its anti-inflammatory and neurorestorative abilities. It is worth to mention that chronic curcumin treatment exhibits distinct effect in bone cancer pain, although the mechanisms are complicated and need to be further investigated.

It was notable that the analgesic effect of curcumin was completely blocked by the non-selective opioid receptor antagonist naloxone (i.p.), suggesting the involvement of the peripheral opioid system in curcumin-induced antinociception. Furthermore, our present data showed that the antinociceptive effect of curcumin was primarily mediated by MOR and DOR receptors. In fact, these are consistent with previous studies demonstrating the opioid mechanism of curcumin in diabetic peripheral neuropathic pain and formalin-induced pain (Tajik et al., 2007; Banafshe et al., 2014). Similarly, Zhao and colleague also found that MOR and DOR receptors are involved in curcumin-induced antinociception in neuropathic pain (Zhao et al., 2012). Previous evidence suggests that opioids can produce antinociceptive effects through peripheral opioid receptors located on sensory neurons (Bartho et al., 1990; Stein, 1995). Opioid receptors are expressed in large-, medium- and small-diameter DRG neurons and then transported to peripheral nerve terminals (Mousa et al., 2001; Stein et al., 2009). Upon activation by opioids, opioid receptors bind to inhibitory G-proteins. This leads to the inhibition of Ca^2+^ or Na^+^ current, and subsequent inhibition of peripheral sensory neurons, which ultimately produces analgesia (Stein et al., 2003). In a variety of preclinical pain models such as inflammatory and neuropathic pain, the opioid receptors in the DRG are up-regulated (Puehler et al., 2006; Ceredig et al., 2019). In addition, opioids produce more significant analgesic effects in injured tissues than in non-injured tissues (Stein et al., 2003; Vanderah et al., 2008). Activating peripheral levels of MOR and DOR can indeed produce significant analgesic activity in cancer-induced bone pain (Baamonde et al., 2005).

Long-term use of opioids can lead to the development of analgesic tolerance. Analgesic tolerance is defined as the decrease in analgesic effect after long-term administration of analgesic drugs, and need to increase the drug dose to maintain the initial analgesic effect (Mercadante et al., 2019). In previous report, morphine-induced peripheral antinociception gradually decreased from day 5 (Zhang et al., 2019). Most strikingly, our results showed that chronic intraperitoneal injection of curcumin has no antinociceptive tolerance in a mice model of cancer-induced bone pain. Unlike exogenous administration of opioids, promoting the release of endogenous opioid peptides often produces non-tolerance forming analgesia. Sodium channel Nav1.7 function loss increased met-enkephalin protein level in mice and humans, resulting in congenital insensitivity to pain (Minett et al., 2015). From another point of view, long-term high levels of met-enkephalin provided intolerable analgesia.

To our knowledge, curcumin is not a direct agonist of opioid receptors. Therefore, combined with the above experimental results, we speculated that endogenous opioid peptides may be involved in the analgesic effect of curcumin. It is well known that there are three main types of endogenous opioid peptides in mammals, namely β-endorphin, enkephalin, and dynorphin. Their precursor genes are *Pomc*, *Penk*, and *Pdyn*. Endogenous opioid peptides are considered to be endogenous ligands of opioid receptors. In detail, β-endorphin has a strong affinity for the MOR receptor, and dynorphin was considered as the endogenous ligand to the KOR receptor (Simon, 1991; Corbett et al., 2006). Enkephalin has a certain degree of excitatory activity on both DOR and MOR receptors, and the selectivity to DOR receptor is higher (Hughes et al., 1975). The endogenous opioid peptides are mainly expressed in the regions involved in the nociceptive response of the central nervous system, including thalamus, limbic system, cortex, periaqueductal gray, and spinal cord (Przewłocki and Przewłocka, 2001). In addition, previous studies found that endogenous opioid peptides are also synthesized by and secreted from DRG neurons and can be transported toward peripheral nerve terminals, mainly demonstrated in cutaneous nerves (Carlton and Coggeshall, 1997; Obara et al., 2009; Stein et al., 2009; Mika et al., 2011). Therefore, we studied the relationship between the analgesic effect of curcumin intraperitoneal injection and the endogenous opioid system in DRG neurons. We found that DRG neurons-expressed β-endorphin and enkephalin were involved in curcumin-induced analgesic effect. Our findings are supported by the following evidence. (1) Treatment with curcumin in cultured primary DRG neurons significantly stimulated the gene expression of *Pomc* and slightly stimulated the gene expression of *Penk* expression but did not achieve a significant difference. (2) Treatment with curcumin in cultured primary DRG neurons specifically stimulated the protein expression of β-endorphin and enkephalin. Interestingly, there was a difference between *Penk* expression level and enkephalin release level induced by curcumin, that is, curcumin promoted the release of enkephalin without affecting the expression of *Penk*. The possible reason is that curcumin only has a regulatory effect on enkephalin at the translational level, but not at the transcriptional level, which is consistent with the differences between the expression of many genes and their corresponding proteins in the organism (Gygi et al., 1999; Greenbaum et al., 2003; Liu et al., 2016c). (3) Moreover, the antiallodynic effect of curcumin was nearly entirely attenuated by the pretreatment with i.pl. administration of β-endorphin antiserum and was partially reduced by enkephalin antiserum. β-endorphin was produced by DRG and transported to peripheral nerve terminals through nerve axons. β-endorphin antibody prevents its binding to opioid receptors, thereby antagonizing its analgesic effect. In fact, previous reports proved that endogenous opioid peptides are involved in the analgesic process of cinobufagin (Apryani et al., 2020), bullatine A (Huang et al., 2016), and cynandione A (Huang et al., 2017), and that the injection of corresponding antibodies can attenuate its analgesia.

Curcumin is known as a safe and non-toxic drug (Soleimani et al., 2018). Therefore, it might be a promising therapeutic option to alleviate cancer pain. A clinical study showed that 6.0 g curcumin administered orally every day for 4–7 weeks during radiotherapy has efficacy against breast cancer patients, with no toxic effects (Ryan et al., 2013). In addition, curcumin has been shown to have both antitumor and analgesic effects, and thus may be beneficial for the treatment of primary tumors. However, curcumin is characterized by low solubility, rapid metabolism, and poor absorption, which greatly limit its clinical usage (Lopresti, 2018). Nelson et al. (2017) even propose that curcumin is a non-bioavailable compound. Therefore, numerous researches have been devoted to improving the bioavailability of curcumin. Various pharmaceutical dosage forms have been developed to overcome these shortcomings, such as solid lipid nanoparticles (Ji et al., 2016), solid dispersions (Li et al., 2015), cyclodextrin inclusion compounds (Rachmawati et al., 2013), liposomes (Guan et al., 2011), and adjuvants (Shoba et al., 1998). However, whether these strategies can be applied in clinical practice needs further study.

## Conclusion

In conclusion, we revealed that intraperitoneal administration of curcumin significantly reduced cancer-induced bone pain symptoms in mice. In addition, curcumin exerted antinociceptive effect by stimulating the expression of *Pomc* and the release of β-endorphin and enkephalin from DRG neurons. These results indicated that curcumin exerted anti-hypersensitivity effect via endogenous opioid peptides, especially β-endorphin and enkephalin, in cancer-induced bone pain. This study, for the first time, clarifies the functional connection between curcumin analgesia and endogenous opioid peptides, supporting the development and potential application of new analgesic drugs based on the structure of curcumin in the treatment of cancer pain.

## Data Availability Statement

The original contributions presented in the study are included in the article/Supplementary Material, further inquiries can be directed to the corresponding author/s.

## Ethics Statement

The animal study was reviewed and approved by the Ethics Committee of Lanzhou University.

## Author Contributions

HZ and KZ conceived and guided the project. GZ, YS, CG, TL, and WN performed experimental work. GZ, LM, ZW, and CD analyzed the data. GZ and HZ wrote the manuscript. All authors contributed to the article and approved the submitted version.

## Conflict of Interest

The authors declare that the research was conducted in the absence of any commercial or financial relationships that could be construed as a potential conflict of interest.

## Publisher’s Note

All claims expressed in this article are solely those of the authors and do not necessarily represent those of their affiliated organizations, or those of the publisher, the editors and the reviewers. Any product that may be evaluated in this article, or claim that may be made by its manufacturer, is not guaranteed or endorsed by the publisher.
