# Supplementary figures and images for: Curcumin Exerts Antinociceptive Effects in Cancer-Induced Bone Pain via an Endogenous Opioid Mechanism
Source: Front Neurosci. 2021 Sep 3;15:696861. doi: 10.3389/fnins.2021.696861 (PMC8446608; doi:10.3389/fnins.2021.696861)

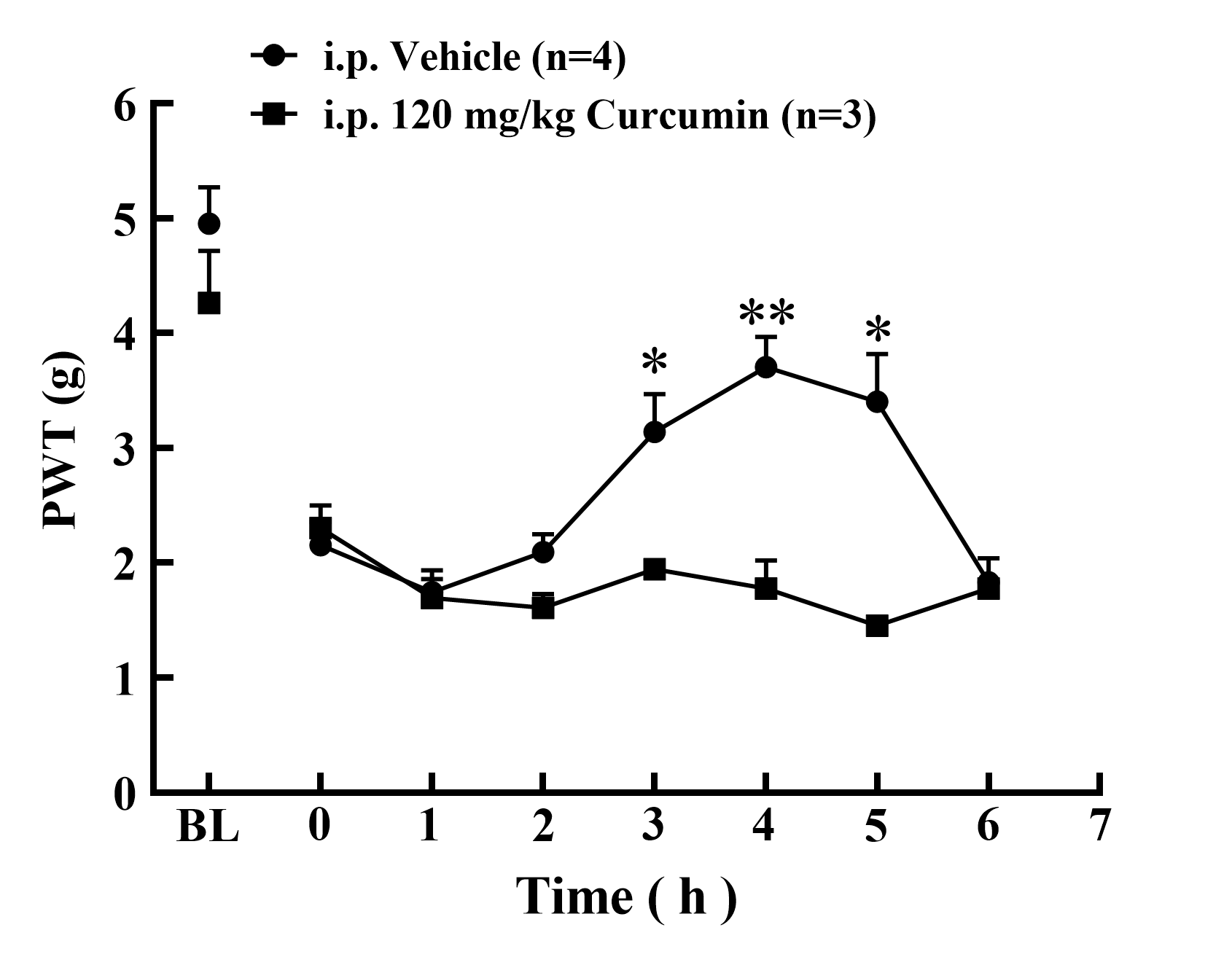

Supplement: Supplementary file 1 [file Image_1.TIF]
